# Haemoglobin concentration and mass as determinants of exercise performance and of surgical outcome

**DOI:** 10.1186/2046-7648-2-33

**Published:** 2013-11-26

**Authors:** James M Otto, Hugh E Montgomery, Toby Richards

**Affiliations:** 1 Division of Surgery and Interventional Science, c/o 4th Floor, Rockefeller Building, 21 University Street, London WC1E 6DE, UK; 2 UCL Institute for Sport, c/o 4th Floor, Rockefeller Building, 21 University Street, London WC1E 6DE, UK

**Keywords:** Total haemoglobin mass, tHb-mass, Haemoglobin concentration, Cardiorespiratory fitness, CPET, Surgical outcome

## Abstract

The ability of the cardiorespiratory system (heart, lungs, blood) to deliver oxygen to exercising skeletal muscle constrains maximum oxygen consumption $\left({\dot{V}O}_{2\max}\right)$, with cardiac output and the concentration of oxygen-carrying haemoglobin ([Hb]) being key limiting parameters. Total blood volume (BV) is the sum of the plasma volume (PV) and the total red cell volume. The measured [Hb] is dependent upon the total circulating mass of haemoglobin (tHb-mass) and plasma volume (PV). While the proportion of oxygen carried in plasma is trivial (0.3 mL of oxygen per 100 mL of plasma), each gram of Hb, contained in red blood cells, binds 1.39 mL of oxygen. As a result, the relationship between ${\dot{V}O}_{2\max}$ and tHb-mass is stronger than that observed between ${\dot{V}O}_{2\max}$ and [Hb] or BV. The glycoprotein hormone erythropoietin drives red cell synthesis and, like simple transfusion of packed red blood cells, can increase tHb-mass. An iron-containing haem group lies at the centre of the Hb molecule and, in situations of actual or functional iron deficiency, tHb-mass will also rise following iron administration. However achieved, an increase in tHb-mass also increases circulating oxygen-carrying capacity, and thus the capacity for aerobic phosphorylation. It is for such reasons that alterations in ${\dot{V}O}_{2\max}$ and exercise performance are proportional to those in arterial oxygen content and systemic oxygen transport, a change in tHb-mass of 1 g being associated with a 4 mL · min^−1^ change in ${\dot{V}O}_{2\max}$. Similarly, ${\dot{V}O}_{2\max}$ increases by approximately 1% for each 3 g · L^−1^ increase in [Hb] over the [Hb] range (120 to 170 g · L^−1^). Surgery, like exercise, places substantial metabolic demands on the patient. Whilst subject to debate, oxygen supply at a rate inadequate to prevent muscle anaerobiosis may underpin the occurrence of the anaerobic threshold (AT), an important submaximal marker of cardiorespiratory fitness. Preoperatively, cardiopulmonary exercise testing (CPET) can be used to determine AT and peak exertional oxygen uptake (${\dot{V}O}_{2}$ peak) as measures of ability to meet increasing oxygen demands. The degree of surgical insult and the ability to meet the resulting additional postoperative oxygen demand appear to be fundamental determinants of surgical outcome: individuals in whom such ability is impaired (and thus those with reduced ${\dot{V}O}_{2}$ peak and AT) are at greater risk of adverse surgical outcome. This review provides an overview of the relationships between [Hb], tHb-mass, exercise capacity, and surgical outcome and discusses the potential value of assessing tHb-mass over [Hb].

## Review

### Introduction

Oxygen (O_2_) must be transported effectively from the atmosphere to the tissues in order to maintain essential metabolic pathways [1]. The heart, vasculature, and blood function to deliver a sufficient supply of O_2_, as well as metabolic substrate, to the tissues to allow effective resynthesis of adenosine triphosphate (ATP) via the electron transport chain (ETC.) [2]. Importantly, O_2_ is the final step in this process acting as the final electron acceptor in the ETC. [3]. Without adequate O_2_ transfer from the blood to the mitochondria, energy-generating mechanisms within the mitochondria would come to a halt [4]. At sites with insufficient O_2_ flow, anaerobic glycolytic metabolism complements ongoing aerobic ATP production, leading to a greater amount of lactic acid [4].

It is generally accepted that the physiological limits of the Fick equation determine the maximal rate at which O_2_ can be transported from the environment to the mitochondria and utilised to support oxidative phosphorylation, termed the maximal oxygen uptake $\left({\dot{V}O}_{2\max}\right)$[5]. This is highlighted in endurance-trained athletes, where O_2_ transport is the most important limiting factor of ${\dot{V}O}_{2\max}$, while mitochondrial O_2_ consumption also limits ${\dot{V}O}_{2\max}$ in untrained individuals [6]. ${\dot{V}O}_{2\max}$ is attained by the simultaneous increase in $\dot{Q}$ (SV × HR) and CaO_2_-CvO_2,_ where $\dot{Q}$ is the cardiac output (determined by the stroke volume (SV) and the heart rate (HR)) and CaO_2_-CvO_2_ is the arteriovenous oxygen content difference. The ability to increase CaO_2_-CvO_2_ depends primarily on the arterial O_2_ content and haemoglobin concentration [Hb] [4].

Haemoglobin is an iron-containing globular protein pigment molecule carried within red blood cells (RBCs) [7]. Haemoglobin carries almost all of the O_2_ in the blood, with a trivial amount dissolved in plasma (0.3 mL O_2_ per 100 mL of plasma) [8]. When fully saturated, assuming a normal [Hb] (e.g. 14 g · dL^−1^ in men) and a constant oxygen capacity of haemoglobin (1.39 mL · g^−1^), haemoglobin carries nearly 20 mL of O_2_ per 100 mL of whole blood [7].

Total haemoglobin mass (tHb-mass) represents the absolute mass of circulating haemoglobin in the body, and can now be quickly, safely, cheaply, and reliably measured using the optimised carbon monoxide (CO) re-breathing method refined by Schmidt and Prommer [9]. Total blood volume (BV) is the sum of plasma volume (PV) and total red cell volume. The measured [Hb] is dependent upon the total circulating mass of haemoglobin (tHb-mass) and plasma volume (PV). However, the proportion of oxygen carried in plasma is trivial, whilst each gram of Hb binds 1.39 mL of oxygen. Thus, tHb-mass largely determines blood O_2_-carrying capacity. In addition, however, tHb-mass can increase BV via its impact on erythrocyte volume [10]. A high BV is essential for achieving a high $\dot{Q}$ as observed in endurance athletes [11,12]. Thus, tHb-mass may be a more sensitive marker of blood O_2_ carrying capacity than using [Hb], and has additional influences (e.g. via impacts on BV) on physical performance than [Hb].

This review provides an overview of the relationships between [Hb], tHb-mass, exercise capacity, and surgical outcome, and discusses the potential value of assessing tHb-mass over [Hb].

### Manipulation of haemoglobin concentration and physical performance

The link between the O_2_-carrying capacity of the blood and indices of exercise capacity such as ${\dot{V}O}_{2\max}$ has a long history. This section will focus on the effects of elevating and reducing [Hb] on markers of cardiorespiratory fitness.

#### Elevation of haemoglobin concentration and maximal oxygen consumption

**Figure 1:**
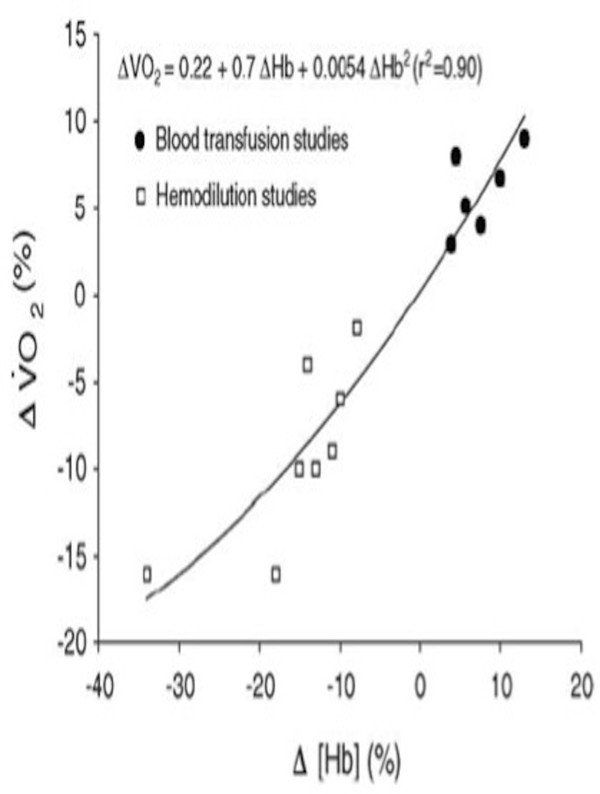
**Relationship between the percent change in [Hb] and percent change in**${\dot{V}O}_{2\max}$**.** Each data point represents the mean of each study using data obtained during the first 48 h after [Hb] manipulation. Figure reproduced with permission from [33] using data from nine studies [14-18,34-37].

${\dot{V}O}_{2\max}$ rises when systemic [Hb] is increased by RBC infusion [13-21] (Figure 1). ${\dot{V}O}_{2\max}$ and/or exercise endurance have also been shown to increase in circumstances where [Hb] has been elevated by the administration of recombinant human erythropoietin (rhEPO) to healthy individuals [22,23], athletes [23,24], haemodialysis patients [25,26], and patients with heart failure [27,28], or through the increased Hb synthesis following administration of iron supplements [29]. Studies that have failed to find such a relationship between [Hb] and exercise capacity [30] may in part be explained by (i) a small quantity of blood being reinfused, (ii) insufficient time for the body to adapt its normal [Hb] post venesection, and (iii) inadequate storage of the RBCs [31]. When these factors are appropriately controlled for, elevating [Hb] is shown to increase ${\dot{V}O}_{2\max}$ and endurance performance [13]. Gledhill and colleagues [31,32] have postulated that ${\dot{V}O}_{2\max}$ increases by approximately 1% for each 3 g · L^−1^ [Hb] over the [Hb] range (120 to 170 g · L^−1^).

#### Reduction of haemoglobin concentration and maximal oxygen consumption

Early work by Ekblom and colleagues [14] demonstrated, in four participants, that a 13% reduction in [Hb] (by venesection of 800 mL of blood) lowered ${\dot{V}O}_{2\max}$ by 10% (from 4.54 to 4.09 L · min^−1^) with a greater effect on endurance time observed (reduced by 30% from 5.77 to 4.04 min). In the same study, an additional four participants underwent sequential venesection of 400, 800, and 1,200 mL of whole blood (at 4-day intervals) that resulted in a reduction in [Hb] of 10%, 15%, and 18%, respectively. These reductions were mirrored by a stepwise impairment in ${\dot{V}O}_{2\max}$ (6%, 10%, and 16% reduction) and endurance times (13%, 21%, and 30% reduction).

Similar findings have been shown by a number of different authors including Balke et al. (9% decrease in ${\dot{V}O}_{2\max}$ 1 h after a 500-mL venesection) [34], Woodson and colleagues (16% decline in ${\dot{V}O}_{2\max}$ after 34% reduction of [Hb]) [35], Kanstrup and Ekblom (9% reduction in ${\dot{V}O}_{2\max}$ and 40% lower endurance time at the intensity eliciting ${\dot{V}O}_{2\max}$ after reducing [Hb] by 11% through the removal of 900 mL blood) [36] and to a lesser extent by Rowell et al. (4% decrease in ${\dot{V}O}_{2\max}$ following a 14% decrease in circulating [Hb] after repeated phlebotomies totaling 700–1,000 mL over 5 days) [37].

#### Change in haemoglobin concentration and anaerobic threshold

Compared to ${\dot{V}O}_{2}$ peak or ${\dot{V}O}_{2\max}$, less is known about the impact of changes in [Hb] on submaximal markers of cardiorespiratory fitness such as the AT. The AT represents the highest ${\dot{V}O}_{2}$ (or running speed, power output) that can be performed without developing a sustained lactic acidosis [38].

Fritsch and colleagues [39] reported CPET in 16 young healthy participants before and 2 days after a 450-mL venesection that resulted in [Hb] being reduced from 14.5 to 13.0 g · dL^−1^ (not classified as anaemic if using the World Health Organisation recommendations [40]). The AT was reduced following venesection when expressed as a percentage of ${\dot{V}O}_{2\max}$ (pre 68.5% versus post 52%) and as an absolute ${\dot{V}O}_{2}$. Our laboratory [41] has shown an independent association between preoperative [Hb] and AT after adjusting ${\dot{V}O}_{2}$ values for known confounders (age, sex, testing site, operation category, diabetes, creatinine) and performing allometric scaling to remove the influence of body size from ${\dot{V}O}_{2}$ values. Causality cannot be conferred from these data, but nonetheless demonstrate that those patients wiot be conferred from these data, but nonetheless demonstrate that those patients with the lowest [Hb] displayed the lowest ${\dot{V}O}_{2}$ values and vice versa. Data from Japan [42] suggest that the AT is lower in patients with iron deficiency anaemia than in non-athletic controls (AT 15.9 ± 3.3 versus 21.3 ± 1.3 mL · kg^−1^ · min^−1^, *p* < 0.01) and responds to increases in [Hb] following iron supplementation ([Hb] 9.0 ± 1.8 to 12.1 ± 0.8 g · dL^−1^), AT (20.9 ± 6.3 to 25.0 ± 8.0 mL · kg^−1^ · min^−1^, *p* < 0.001).

### Relationship between tHb-mass, blood volume, and exercise capacity

**Figure 2:**
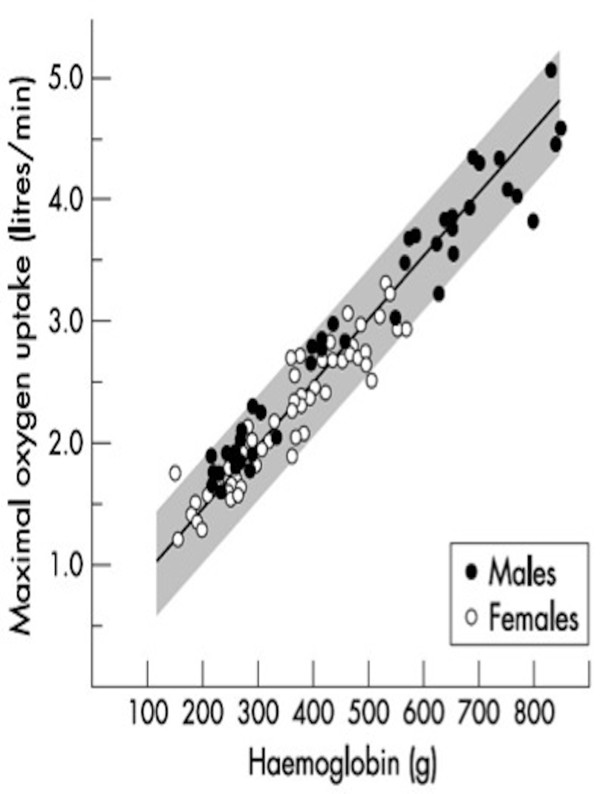
**Relationship between total body haemoglobin (between 100 and 900 g) and**${\dot{V}O}_{2\max}$**in 94 individuals aged 7–30 years**[45]**.** Figure reproduced with permission from [46].

The relationship between markers of cardiorespiratory fitness and tHb-mass is stronger than that with BV or [Hb] [43,44]. A high correlation between tHb-mass and ${\dot{V}O}_{2\max}$ (*r* = 0.97) was observed in the early 1950s by Astrand [45], where differences in maximal aerobic capacity between adults and children and between men and women were related to differences in total haemoglobin (see Figure 2). This initial investigation laid the foundation for much of the subsequent work in relation to tHb-mass and aerobic capacity.

Subsequently, undertaking a meta-analytical approach, Schmidt and Prommer [43] pooled data from 611 subjects. ${\dot{V}O}_{2\max}$ was determined using either an incremental cycle ergometry test or treadmill protocol, with. values obtained from treadmill exercise adjusted (specifically reduced) by 7% to account for the greater muscle mass utilised compared to cycling. tHb-mass was measured in all subjects using the CO re-breathing technique. Results revealed a high correlation (*r* = 0.79) between ${\dot{V}O}_{2\max}$ and tHb-mass. A similar close dependency between BV and ${\dot{V}O}_{2\max}$ (*r* = 0.76) was highlighted, in keeping with early work by Convertino that showed a similar relationship between total BV and ${\dot{V}O}_{2\max}$ (*r* = 0.78) [47]. No significant dependency of ${\dot{V}O}_{2\max}$ on [Hb] (males *r* = 0.03, females *r* = 0.12) or Hct (males *r* = 0.08, females *r* = 0.11) was observed.

A number of other cross-sectional studies have demonstrated a strong positive association between ${\dot{V}O}_{2\max}$ and tHb-mass including that by Gore and colleagues [48] who studied a cohort of trained athletes, female rowers (n = 17, r = 0.92, p < 0.0001), male rowers (*n* = 12, *r* = 0.79, *p* < 0.005) and male runners (*n* = 33, *r* = 0.48, *p* = 0.005). Likewise, Heinicke et al. [49] investigated BV and tHb-mass in elite athletes of different disciplines (downhill skiing, swimming, running, triathlon, cycling junior, and cycling professional), finding that ${\dot{V}O}_{2\max}$ was significantly related to tHb-mass not only in the whole group but also in all endurance disciplines.

### Changes in tHb-mass and exercise capacity

Procedures to increase tHb-mass result in elevated ${\dot{V}O}_{2\max}$, whereas the opposite is true when tHb-mass is reduced [36], highlighting the importance of tHb-mass as a primary determinant of ${\dot{V}O}_{2\max}$ by determining O_2_-carrying capacity.

#### Elevation of tHb-mass and exercise capacity

When tHb-mass is increased through the use of rhEPO, concomitant increases in ${\dot{V}O}_{2\max}$ have been reported. Specifically, ${\dot{V}O}_{2\max}$ increased by 6%–7% in 27 recreational athletes after an increase in tHb-mass of 7%–12% and both fitness and blood parameters returned to baseline after cessation of rhEPO [50]. Similarly, a recent study in 19 trained men showed an improved 3,000-m running time trial performance (11:08 ± 1:15 to 10:30 ± 1:07 min/sec, *p* < 0.001) following 4 weeks of rhEPO administration. This improved performance coincided with a rhEPO-induced increase in ${\dot{V}O}_{2\max}$ (56.0 ± 6.2 to 60.7 ± 5.8 mL · kg^−1^ · min^−1^, *p* < 0.001) and tHb-mass (12.7 ± 1.2 to 15.2 ± 1.5 g · kg^−1^, *p* < 0.001).

What change in aerobic capacity can we expect for a given change in tHb-mass? Linear regression analysis revealed a change in tHb-mass of 1 g · kg^−1^ was associated with a change in ${\dot{V}O}_{2\max}$ of 4.4 mL · kg^−1^ · min^−1^ (males 4.2 mL · kg^−1^ · min^−1^, females 4.6 mL · kg^−1^ · min^−1^) and a change in BV of 1 mL blood per kilogram was related to a change in ${\dot{V}O}_{2\max}$ of 0.7 mL · kg^−1^ · min^−1^[43]. In 144 male athletes of various specialities with absolute ${\dot{V}O}_{2\max}$ values ranging from 1,010 to 6,320 mL · min^−1^ and tHb-mass from 242 to 1,453 g, a change in 1 g of haemoglobin was associated with a change in ${\dot{V}O}_{2\max}$ by around 4 mL · min^−1^[51]. This is the same as reported by Gore and colleagues [48] and very similar to that recently reported in an excellent review article in this area [10]. Understanding what change in aerobic capacity we can expect from a change in tHb-mass is important because it allows an accurate prediction of likely improvements in functional capacity as a result of an intervention to improve tHb-mass.

#### Reduction of tHb-mass and exercise capacity

After 550 mL of whole blood had been withdrawn from 9 moderately trained male and female athletes, tHb-mass was reduced on average by 77 ± 21 g [52]. This was significantly associated with a decline in ${\dot{V}O}_{2\max}$ of 255 ± 130 mL · min^−1^ (1 day post phlebotomy) and was still decreased on day 10 (197 ± 116 mL · min^−1^). The authors commented on a suppression of endurance performance during this period of lower tHb-mass. tHb-mass has also been shown to be reduced (868 ± 99 to 840 ± 94 g, *p* = 0.03) following a 30-day detraining period (87% reduction in training hours) with a reciprocal decrease in ${\dot{V}O}_{2\max}$ (4.83 ± 0.29 to 4.61 ± 0.41 L · min^−1^) observed [53]. Given these findings and that tHb-mass is lower in healthy sedentary individuals than in those who are athletically trained [54], would sick patients have a lower tHb-mass by virtue of inactivity? And might the relationship between lower aerobic capacity and poorer operative outcome be in part mediated through a sedentary lifestyle-associated reduction in tHb-mass?

### Mechanisms for reduced exercise capacity following haematological changes

A reduction in [Hb] due to a fall in tHb-mass may impair exercise capacity in a number of ways. Firstly, a reduction in CaO_2_ will reduce muscle O_2_ availability (O_2_ delivery) for the same muscle blood flow [55]. Secondly, muscle O_2_-diffusing capacity is lower when [Hb] is reduced, which may be related to alterations in the intracapillary spacing of erythrocytes or slower dissociation of O_2_ from [Hb] [56]. Thirdly, pulmonary diffusion is reduced when [Hb] is reduced. Finally, a reduction in circulating BV may also impact aerobic capacity by affecting ventricular preload (diastolic function) via the Frank-Starling mechanism, thus altering SV and $\dot{Q}$[11,57]. However, it appears that the predominant mechanism explaining the detrimental impact of reduced [Hb] on ${\dot{V}O}_{2\max}$ and (to a greater extent) exercise endurance is the lowered O_2_-carrying capacity of the blood [33], with [Hb] being more important to ${\dot{V}O}_{2\max}$ in the untrained than in trained individuals [6]. This may have significant implications in patient populations.

Similar mechanisms may underpin the reduced AT observed when [Hb] is reduced but this is a much-debated and controversial concept [58,59]. The AT represents the highest ${\dot{V}O}_{2}$ (or running speed, power output) that can be performed without developing a sustained lactic acidosis [38]. When performing exercise above the AT, it is suggested that the metabolic demands of tissues (mitochondria) outstrips O_2_ supply, and aerobic ATP resynthesis is supplemented by anaerobic metabolism leading to increased lactate production relative to the rate of glycolysis (i.e. increased lactate/pyruvate ratio) [60]. The AT is therefore an important marker of cardiorespiratory fitness as it provides an assessment of the ability of the cardiovascular system to supply O_2_ at a rate adequate to prevent muscle anaerobiosis [38]. A reduced capacity to supply O_2_ to actively respiring tissues caused by low [Hb] or cardiovascular disease conditions has the potential to reduce the AT.

### Surgical outcome, tHb-mass, and cardiorespiratory fitness

The measurement of tHb-mass (rather than [Hb]) in the clinical setting may have important applications but these remain relatively unexplored. For example, [Hb] may vary as intravascular fluid shifts as a result of disease states or their treatment, making it a poor index of oxygen-carrying capacity. [Hb] is determined by tHb-mass and the total volume of blood. A substantial reduction in oxygen-carrying capacity, related to a low tHb-mass, may thus be masked if PV is contracted, as may be the case in many disease states. Similarly, increases in intravascular volume may depress [Hb], even in the context of a normal tHb-mass. Knowledge of tHb-mass and [Hb] allows calculation of PV as a separate variable, allowing evaluation of disease-related fluid shifts. The degree of surgical blood loss might also be better quantified through the measurement of tHb-mass than [Hb]. More importantly, perhaps, tHb-mass may represent a more sensitive marker of blood O_2_ transport capacity than [Hb] in isolation [61].

Major surgery can be defined as any intervention occurring in a hospital operating theatre involving the incision, excision, manipulation, or suturing of tissue, usually requiring regional or general anaesthesia or sedation [62]. The determinants of surgical outcome (morbidity and mortality) are related to an interplay between the health and fitness of patients, the number and severity of comorbidities present [63], and patient age as well as surgery-related factors (emergency or planned, mode, type, and duration). In addition, the systemic inflammatory response caused by hormonal, immunological, and metabolic mediators [64] is essential for effective tissue repair and healing after surgery. Effective O_2_ delivery to the tissues during the hypermetabolic postoperative period is thought to be a fundamental determinant of surgical outcome [65,66] with patients who are unable to raise O_2_ delivery to meet the increased ${\dot{V}O}_{2}$ requirement more frequently developing complications [67,68]. The cause of this uncoupling of O_2_ supply and demand is multifactorial but may be predominantly linked to the interaction between a patient's existing comorbidities (e.g. cardiac disease, respiratory disease, or indeed any condition that impairs O_2_ delivery and/or cardiac output) and the degree of surgical insult [69].

Impairment in the ability to meet these demands can be determined preoperatively through the assessment of exertional ${\dot{V}O}_{2}$ peak and AT (by CPET); reductions in both markers of functional capacity are associated with an increased risk of perioperative morbidity and mortality [70-74]. The original work by Older and colleagues almost 2 decades ago was the first to highlight the association between low functional capacity by CPET and adverse patient outcome following non-cardiopulmonary surgery [75]. Specifically, a reduced cardiorespiratory reserve, typically defined as an AT of less than 11 mL · kg^−1^ · min^−1^ being associated with an increased risk of adverse postoperative outcome following major intra-cavity surgery [74]. Similarly, impaired ${\dot{V}O}_{2}$ peak has been shown to predict worse postoperative outcome following major lung resection (${\dot{V}O}_{2}$ peak <20 mL · kg^−1^ · min^−1^[76], <15 mL · kg^−1^ · min^−1^[77]) and bariatric surgery (${\dot{V}O}_{2}$ peak <16 mL · kg^−1^ · min^−1^) [78]. The reader is referred to an excellent systematic review in this area covering the role of CPET as a preoperative risk stratification tool in non-cardiopulmonary surgery for more details [74].

It is acknowledged that although the ${\dot{V}O}_{2}$ response from an exercise test is not directly comparable to that in a postoperative patient, common with exercise, ${\dot{V}O}_{2}$ postoperatively in major surgery is high [79]. For example, preoperative resting ${\dot{V}O}_{2}$ has been shown to increase from 110 to approximately 170 mL · min^−1^ · m^−2^[80,81] indicating a greater requirement for O_2_ following surgery. In this context, tHb-mass may be important to surgical outcome due to its role in determining O_2_ delivery. This may be related to the close linear relationship that exists between tHb-mass, BV, $\dot{Q}$, and aerobic capacity [10]. For example, a high BV is a prerequisite for a high tHb-mass, which in turn impacts upon $\dot{Q}$ by elevating venous return and cardiac filling pressures [82,83]. Because tHb-mass in combination with BV also governs [Hb] and therefore oxygen-carrying capacity, the effects of tHb-mass on determining O_2_ delivery are twofold. Given the close relationship between tHb-mass and aerobic capacity and the association between markers of cardiorespiratory fitness (${\dot{V}O}_{2}$ peak and AT) and surgical outcome, it would seem intuitive that a high tHb-mass may confer a survival advantage in the perioperative setting. If this is the case, then strategies aimed at elevating tHb-mass may improve outcome (morbidity and mortality) following surgery, but this remains to be confirmed. Given that anaemia is associated with an increased risk of adverse surgical outcome, it would be surprising if this relationship were not maintained for tHb-mass.

## Conclusion

Changes in [Hb] and tHb-mass are associated with reciprocal alterations in exercise capacity proportional to the change in oxygen-carrying capacity of the blood. tHb-mass displays a stronger relationship with ${\dot{V}O}_{2\max}$ than [Hb] or BV. In the context of surgery, patients with an inability to raise oxygen delivery to meet the increased ${\dot{V}O}_{2}$ requirement of the perioperative period will more frequently develop complications. Impairment in the ability to meet these demands can be determined preoperatively through the assessment of exertional ${\dot{V}O}_{2}$ peak and AT (by CPET), reductions in both markers being associated with an increased risk adverse surgical outcome. Whether differences in tHb-mass are associated with postoperative outcome is not known but an interesting question given the high prevalence of preoperative anaemia itself being associated with an increased risk of poor outcome. In addition, the extent to which postoperative outcomes are dependent upon interactions between [Hb], tHb-mass, and ${\dot{V}O}_{2}$ is unknown and whether strategies to increase tHb-mass result in improved surgical outcome remains to be clarified.

## Abbreviations

AT: anaerobic threshold; CaO2-CvO2: arteriovenous oxygen content difference; BV: blood volume; CO: carbon monoxide; CPET: cardiopulmonary exercise testing; rhEPO: recombinant human erythropoietin; ESA: erythropoietin-stimulating agent; EV: erythrocyte volume; DO2: oxygen delivery; Hct: haematocrit; Hb: haemoglobin concentration; O2: oxygen; PA: physical activity; PV: plasma volume; tHb-mass: total haemoglobin mass; ${\dot{V}O}_{2}$: oxygen consumption; ${\dot{V}O}_{2\max}$: maximal oxygen consumption; ${\dot{V}O}_{2}$ peak: peak oxygen consumption; CaO2: arterial oxygen content; $\dot{Q}$: cardiac output.

## Competing interests

JMO is receiving an Impact PhD Studentship part-funded by VIFOR (INTERNATIONAL) Inc. with a total funding of £32,534 over 3 years. All remaining authors declare that they have no competing interests.

## Authors’ contributions

JMO, HEM, and TR were responsible for drafting and revising the article. All authors read and approved the final manuscript.
